# Carbon Fluxes and Stocks by Mexican Tropical Forested Wetland Soils: A Critical Review of Its Role for Climate Change Mitigation

**DOI:** 10.3390/ijerph17207372

**Published:** 2020-10-09

**Authors:** Sergio Zamora, Luis Carlos Sandoval-Herazo, Gastón Ballut-Dajud, Oscar Andrés Del Ángel-Coronel, Erick Arturo Betanzo-Torres, José Luis Marín-Muñiz

**Affiliations:** 1 Faculty of Engineering, construction and Habitat, Universidad Veracruzana Bv. Adolfo Ruíz Cortines 455, Costa Verde, Boca del Rio C.P., Veracruz 94294, Mexico; szamora@uv.mx; 2 Division of Research, Postgraduate Studies and Innovation, Tecnológico Nacional de México/Instituto Tecnológico Superior de Misantla, Veracruz C.P., Misantla 93821, Mexico; lcsandovalh@gmail.com (L.C.S.-H.); gballut@hotmail.com (G.B.-D.); erickbetanzo@hotmail.com (E.A.B.-T.); 3 Facultad de Ingeniería, Universidad de Sucre, Cra. 28 #5-267, Puerta Roja, Sincelejo, Sucre 700001, Colombia; 4 Tecnológico Nacional de México/InstitutoTecnológico Superior de Huatusco, Programa de Maestría en Ingeniería, AV. 25 Poniente N° 100, Col Reserva Territorial, Huatusco, Veracruz 94100, Mexico; oscardelangel.coronel@hotmail.com; 5 Academy of Sustainability and Regional Development, El Colegio de Veracruz, Xalapa, Veracruz 91000, Mexico

**Keywords:** forested wetlands, carbon pool, ecosystem services, carbon sequestration

## Abstract

Wetland soils are important stores of soil carbon (C) in the biosphere, and play an important role in global carbon cycles in the response strategy to climate change. However, there areknowledge gaps in our understanding of the quantity and distribution in tropical regions. Specifically, Mexican wetlands have not been considered in global carbon budgets or carbon balances for a number of reasons, such as: (1) the lack of data, (2) Spanish publications have not been selected, or (3) because such balances are mainly made in the English language. This study analyzes the literature regarding carbon stocks, sequestration and fluxes in Mexican forested wetlands (Forest-W). Soil carbon stocks of 8, 24.5 and 40.1 kg cm^−2^ were detected for flooded palms, mangroves, and freshwater or swamps (FW) wetland soils, respectively, indicating that FW soils are the Forest-W with more potential for carbon sinks (*p* = 0.023), compared to mangroves and flooded palm soils. While these assessments of carbon sequestration were ranged from 36 to 920 g-C m^−2^ year^−1^, C emitted as methane was also tabulated (0.6–196 g-C m^−2^ year^−1^). Subtracting the C emitted of the C sequestered, 318.2 g-C m^−2^ year^−1^ were obtained. Such data revealed that Forest-W function is mainly as carbon sink, and not C source. This review can help to inform practitioners in future decisions regarding sustainable projects, restoration, conservation or creation of wetlands. Finally, it is concluded that Forest-W could be key ecosystems in strategies addressing the mitigation of climate change through carbon storage. However, new studies in this research line and public policies that protect these essential carbon sinks are necessary in order to, hopefully, elaborate global models to make more accurate predictions about future climate.

## 1. Introduction

Wetlands are the ecotones or transitional zones between permanently aquatic and dry terrestrial ecosystems. They are among the most productive ecosystems, are found in almost all parts of the world, and their most notable features are the presence of standing water for some period during the growing season, unique soil conditions, organisms, and vegetation [1,2,3,4].

Some ecosystem services (the ecological processes that natural ecosystems provide humanity with a large and important range of free services on which we depend) provided by wetlands include: water purification, climate regulation, they have been found to cleanse polluted water, protection of shorelines, flood regulation, and are described as the kidney of the planet [2,3]. They also provide a potential or carbon pool for atmospheric carbon but, if not managed properly, they may become a source of greenhouse gases (GHGs). The Intergovernmental Panel on Climate Change of the United Nations hasindicated that carbon sequestration is a low-cost alternative to reduce atmospheric carbon dioxide [4]. Carbon is sequestered in wetlands when C inputs (productivity and/or sedimentation) surpasses C outputs (decomposition and C exports), and the remaining organic material, mostly senesced plant material, is accumulated in the wetland’s anaerobic sediment layer as a mat of partially decayed organic material [4,5,6]. In addition to GHGs emitted as a result of anthropogenic activities, almost one-third of GHGs emission is from natural sources such as wetland soils. Field studies have found that GHGs emissions in wetland soils are controlled by physical factors such as temperature, hydrology, and vegetation type [7,8].

Forested wetlands are a type of wetlands dominated by trees or shrubs, according to the U.S. definition [1]. Other definitions argue that Mexican forested wetlands in coastal are also dominated of palms; in this case, flooded palms [9]. These wetlands are the most common type of wetlands along the Mexican coast and, according to their water type, they may include mangroves in brackish water and freshwater wetlands with trees or palms. Some studies have reported that the rate of carbon sequestration is higher in forested wetland soils than marshes [7,10]. Forested wetland systems, though much smaller in size than the planet’s forests, sequester this carbon at a much faster rate, and can continue to do so for millions of years. Most of the carbon taken up by these ecosystems is stored below ground where we cannot see it, but it is still there. When the carbon captured is by the world’s ocean and coastal ecosystems, it is denoted “blue carbon”, acting as carbon sink or carbon pool. One method to slow climate change impacts is to incorporate coastal wetlands into the carbon market through the buying and selling of carbon offsets. By means of this, a financial incentive for restoration and conservation projects may be created by helping to alleviate federal and state carbon taxes aimed at discouraging the use of fossil fuels. When fewer greenhouse gases are emitted, less pollution is created. When there is less pollution to tax, the process benefits not only the environment, but also the financial well-being of the community doing the restoration [11].

Hansen [12] reported that from 20% to 35% of the forested wetlands of the Mississippi alluvial valley and Gulf-Atlantic coastal flat regions could have carbon offset values that exceeded the cost of restoring the wetland and the opportunity cost of moving the land out of agricultural production. Wetlands cover approximately 6% of the Earth’s land surface [1], which is approximately similar to 244,794,979 ha of wetland area around the world [13]. Almost 56% of this estimated total wetland area is found in tropical and subtropical regions like Mexico [1]. Olmsted [14] estimated that there are more than 3.3 million hectares of wetlands in Mexico, (approximately 0.6 % of the world’s total wetlands). However, using the available maps, as well as the digital elevation model (NASA), it was estimated that more than half of the Mexican states that have wetlands have lost more than 50% of them [15]. It is essential to know the importance of carbon sinks of wetlands in order to be considered in global carbon budgets and projects of restoration, creation and conservation of wetland ecosystems. The few existing carbon balances include data mainly from boreal and temperate wetlands [16,17,18,19], due to the scarcity of data from tropical regions such as Mexico. The objective of this study is to quantify the function as carbon sink in coastal Mexican forested wetlands according to the literature, compare the carbon pool among palms, mangroves and freshwater wetland soils, typical forested wetlands from Mexico and analyze the importance of mitigation of climate change with tropical forested wetlands through carbon stocks.

## 2. Materials and Methods

The authors undertook a comprehensive search of the literature on carbon dynamic in Mexican forested wetland soils (mangroves, flooded palms, and freshwater wetlands) based on the most important databases located in Mexican universities such as Universidad Nacional Autónoma de México (UNAM), Universidad Autónoma Metropolitana (UAM), Colegio de Postgraduados (COLPOS), Universidad Veracruzana (UV), El Colegio de Veracruz, Instituto de Ecología A.C., publications of the Mexican carbon program (http://pmcarbono.org/pmc/publicaciones/sintesisn.php), and the ISI Web of Knowledge (www.isiknowledge.com) database. The keywords used were: carbon (pool, stock, sinks, sequestration, fluxes), soil, mangroves, freshwater, forested, palm, and swamp (wetlands) (exclusively in Spanish and English). A total of 285 studies (from the year 2000 to 2020) were identified regarding carbon fluxes and carbon sinks for Mexican wetland soils; only 12% were selected (34 studies) based on studies about carbon fluxes in forested Mexican wetlands. The remaining percentage of studies was used for introduction questions, justification of the study and discussion of the data.

Statistical analyses to determine differences among wetland soilcarbon stocks were performed with IBM SPSS Statistic version 22 for Windows (Armonk, NY, USA: IBM Corp.). Kruskal–Wallis test at 5% significantlevel was also used.

## 3. Results

The importance of wetlands to the global carbon cycle and ecosystem services is generally known, but the extent to which they affect (carbon source or sink) the carbon cycle is poorly understood. Wetlands may affect the atmospheric carbon cycle in four ways. Firstly, many wetlands have highly labile carbon and these wetlands may release it if water level is lowered or management practices result in oxidation of soils (it involves aerobic and anaerobic processes) [1,5,20]. Secondly, the entrance of carbon dioxide into a wetland system is via photosynthesis by wetland plants, giving it the ability to alter its concentration in the atmosphere by fixing this carbon in the soil [2,5,6]. Thirdly, wetlands are prone to trap carbon rich sediments from watershed sources and may also release dissolved carbon into adjacent ecosystems. This, in turn, may affect both sequestration and emission rates of carbon [1,6,20]. Lastly, wetlands are also known to contribute in the release of methane to the atmosphere, even in the absence of climate change [7,16,17].

The importance of wetlands protection has been receiving heightened attention because of recognition of their high ecosystem carbon stocks; such function can be a pathway to help ameliorate greenhouse gas emissions. However, few studies in tropical Mexican wetlands have quantified ecosystem carbon stock (carbon sequestered in the soil by area) or carbon sequestration (carbon sequestered in the soil by area and time). In studies about carbon balance in wetlands in the world [1,17,18,19,20], generally Mexican wetlands are not considered by the lack of information or because such data are in Spanish, in this review, we described some studies (Table 1) that showed the importance of Mexican forested wetlands in carbon stock in the soil.

**Figure 1:**
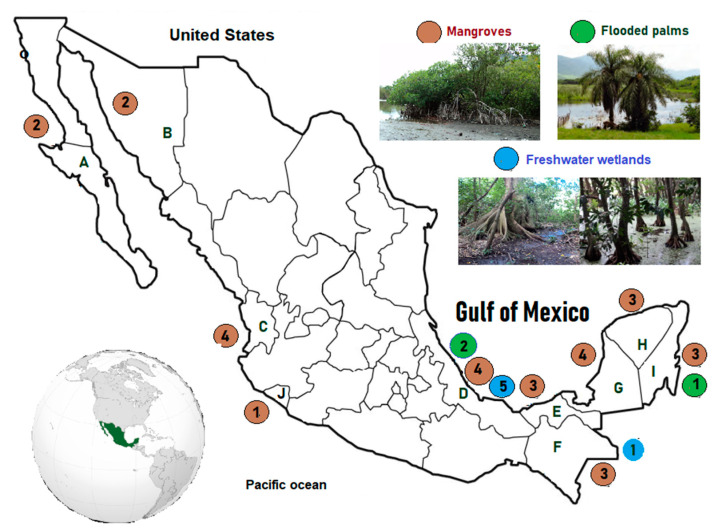
Location of the forested wetlands reviewed. Places represented by letters are referenced in Table 1 and Table 2. The number inside the circle is the number of studies in that state/site.

In Mexico, studies of carbon stock and fluxes in wetland soils have focused mainly on brackish wetlands (mangrove ecosystems) [4,21,22,23,24,25,26,27,28,29,30,31,32,33,34,35,36,37,38,39,40,41] (Table 1, Figure 1). Mangrove forests in Mexico are dominated mainly by four species: red mangrove (*Rhizophora mangle*), white mangrove (*Laguncularia racemosa*), black mangrove (*Avicennia germinans*), and button wood mangrove (*Conocarpus erectus*). For such wetland types, carbon stock values of 7.9 to 65 kg C m^−2^ were reported; differences in carbon stock are related with the depth of soil measured. Carbon stocks observed in Mexican mangroves soils were higher than the carbon stocks reported on tropical mangroves of Kerela, India (13.9 kg C m^−2^) [47], Sofala, Mozambique (15.9 kg C m^−2^) [48] and Honduran mangroves (5.7–10.6 kg C m^2^) [49]. Despite its good storage of carbon in the soil of Mexican mangroves, its extent has decreased almost 55% from 1970 to 2018 (1,420,000 ha to 775,555 ha) [50], mainly by land use change or conflict between cattle ranches and fishermen in mangrove areas [51]. Owing to this, projects for reforestation and restoration of mangroves are necessary. In 2018, the approved reforms to the General Law on Climate Change (LGCC) aligned the Mexican law with the international objectives established in the 2nd Article of the Paris Agreement. This action proves Mexico’s commitment to contributing to the global target of stabilizing the GHGs emissions concentration in the planet. Thus, restoring and conserving mangroves for carbon sequestration or carbon pool could contribute to fulfilling this commitment.

On the other hand, FW and flooded palms are ecosystems with recent interest in knowing the carbon pool function, the number of publications and study sites on carbon dynamics in Mexican FW (six studies; Table 1) and palm flooded (three studies: Table 1) isstill very small. However, the evaluations revealed that carbon stock in FW is similar (9.5–60 kg C m^−2^; Table 1) that reported for mangroves (7.9 to 65 kg C m^−2^). In Mexico, mangroves are among the species with environmental protection because they are threatened species and are therefore protected by the Mexican law (NOM-059-ECOL-2001) [2]. However, laws for freshwater wetlands have not been established yet; the data showed in Table 1 reveals the importance of carbon stock as ecosystem services similarly to mangroves. Thus, it is necessary to implement similar legality for FW as with mangroves.

The lack of public policies to protect the FW has caused land use change, mainly from tropical wetlands to pastures for the introduction of cattle [52], the degree of impact depends on the number of cows, the time they are in the wetland transformed, and modifications to hydroperiod and vegetation. Comparing the carbon stock observed for tropical Mexican FW with the C stocks in other sites, this is up to 50% larger than observed for FW in Pennsylvania, 7 to 30 kg C m^−2^ were reported [53]. Similarly, 11−29 kg C m^−2^ were detected for alpine and other FWs in south-eastern Australia [54]. Carbon storage in ranges from 2 to 20 kg C m^−2^ were reported in FW of South Korea [55]. The above shows the importance of conserving and creating new FW. With the data that we documented, a measure that helped to enhance carbon storage in soil and therefore enable these ecosystems remains vital in global carbon balance and climate change mitigation.

A study using a dynamic model that includes productivity, respiration of plant and soil, carbon sequestration, gas fluxes, the half-life of the gases in different time horizons with data of FW by Marín-Muñiz and Hernández [56], showed that FW should be considered as sinks of carbon in time horizons within 100 years. Additional studies of carbon balance that include mangrove, FW and palm swamps are necessary for a better understanding on how they differ from carbon balance in wetlands from other countries, continents, or regions, and should be incorporated into global climate change models.

The flooded palm soils are less evaluated about the carbon stock function; however, the values reported (1.5–16 kg C m^−2^) are comparable with values reported for some FW and mangrove ecosystems (Table 1). In addition, palms are a resource of great value in the tropics; five species were reported in Mexican wetlands (*Coco nucifera*, *Sabal mexicana*, *Attalea liebmannii*, *Roystoneadun lapiana*, and *Acrocomia aculeata*), the main uses for the five species were for food and construction material, knowing such uses and the carbon pool function, palm cultivation, and reforestation projects should be encouraged and implemented [57,58], the same authors described that it is important to recover and promote the traditional use and value of palm trees, especially for the native species, because of both the economic benefits and the ecosystem services they provide, including carbon pool function. In sum, more participatory work with the inhabitants is needed to initiate palm breeding programs to assist in the recovery of wetland ecosystems.

**Figure 2:**
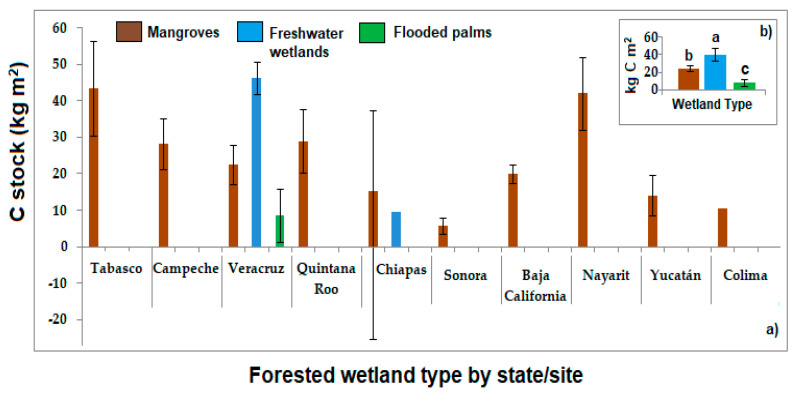
Carbon stock in Mexican forested wetlands by states (**chart a**) and average by wetland type (**chart b**). Values shown in bars are mean. Lines over the bars are the standard error. Letters over the bars of **chart b** represents statistical analysis (different letters imply values significantly different (*p* < 0.05) from each other).

With the same data reported in Table 1, the average values of carbon stock were grouped in the three forested wetland types (FW, mangroves, and flooded palms) by the ten states of Mexico, with more data analyzed and represented in Figure 2a,b, where it is noted that in the State Veracruz there are more wetland sites studied, including mangroves, flooded palms, and FW. In Chiapas, for instance, there are studies of carbon stocks of mangroves and FW. The other sites only present studies of mangroves. In Figure 2b, data were represented by average between three forested wetland types, revealing a significant difference of carbon stock according to the wetland (*p* < 0.05). Significantly higher carbon stock in FW (40.1 ± 7.1 kg C m^−2^) than in mangroves (24.5 ± 3.0 kg C m^−2^) (*p* = 0.027) and flooded palm wetland (8.0 ± 4.3 kg C m^−2^) (*p* = 0.039) was detected. More studies about carbon stock in flooded palms are necessary to have a better panorama of this forested wetland type role on carbon cycle and climate change mitigation. It is important to underline that the values of carbon stocks founded in Mexican forested wetlands were higher than other reported for temperate forested wetlands in Ohio, USA (10.8 kg C m^2^) [59], or than carbon stock reported for floodplain wetlands from Okavango Delta, Botswana (0.8–1.5 kg C m^−2^) [60], or forested wetlands in Costa Rica (1.2–1.6 kg C m^−2^) [59]. On the other hand, Vega-López [61] reported that the carbon stock for terrestrial ecosystems was 6.2 kg C m^2^, a value almost seven times lower than Forest-W, while in marshes the carbon stock in soils was 31.9., indicating the relevance of tropical Mexican forested wetland conservation, creation and reforestation to maintain and increase the carbon pool function of wetlands.

Besides carbon stocks, it is important to know the annual carbon accumulation in the forested wetlands, less studies report it because it is necessary to measure the accretion rate (soil accumulated during a defined period of time–cm year^−1^), data that together with organic carbon and bulk density are used to obtain carbon sequestration (g-C m^−2^ year^−1^) [62]. The assessments of carbon sequestration in Mexican forested wetlands have been showed in Table 2, where the range is between 36 and 920 g-C m^−2^ year^−1^. The higher values were reported for freshwater forested wetlands (920 g-C m^−2^ year^−1^) followed by mangroves (38 g-C m^−2^ year^−1^), and flooded palm wetlands (45 g-C m^−2^ year^−1^). In natural tropical wetlands of Botswana and Costa Rica, values from 3 to 9 times lower (100 to 306 g-C m^−2^ year^−1^) than those measured from freshwater forested Mexican wetlands, were reported [19,59,63]. Similar values were reported in temperate forested wetland sites (180–280 g-C m^−2^ year^−1^) [19,64,65], while in boreal forested wetlands, carbon sequestration values were reported from 15 to 29 g-C m^−2^ year^−1^ [66,67]. Some authors [66,68] pointed out the highly recalcitrant character of the organic matter contained in tropical ecosystems, much more recalcitrant than in boreal wetlands, since labile plant debris (e.g., leaves) decompose very quickly in warm and humid climates, where biologically active C (i.e., microbial communities) is much more active than in colder climates. Previous data revealed the importance of Mexican Forest-W in carbon sequestration environmental service.

Regarding carbon emitted by methane gas, 11 to 196 g-C m^−2^ year^−1^ have been reported for Mexican wetlands, which are values similar to those reported for forested wetlands in other tropical (19–263 g-C m^−2^ year^−1^) and temperate (5–102 g-C m^−2^ year^−1^) regions [19,69,70]. However, the gas emission in tropical wetland soils was higher than in wetlands from boreal ecosystems (1–36 g-C m^−2^ year^−1^) [17,71,72], related with the cool temperature over boreal wetlands that reduces plant productivity and decline in the methane fluxes (a temperature effect). Averaging the carbon sequestration (392 g-C m^−2^ year^−1^), and subtracting the carbon emitted reported by methane (73.8 g-C m^−2^ year^−1^), 318.2 g-C m^−2^ year^−1^ revealed the differences in carbon fluxes from Mexican forested wetlands.

**Table 2:** Carbon fluxes (carbon sequestration and carbon emitted as methane) in Mexican forested wetland soils based on field studies.

Wetland Type/Site	Carbon Sequestration (g-C m^−^^2^ year^−^^1^)	Methane Emissions (g-C m^−^^2^ year^−^^1^)	Location in the Map (Figure 2)	Study Period	References
Mangrove Palm FW	38 45 920		D	*	Moreno-Casasola et al. [27]
Mangroves		<1	D	1 year	Hernández and Junca-Gómez [33]
Tidal wetlands with forest and marsh species mixed.	36.5		A	*	Burke and hinojosa [73]
FW		13.9	E	1 year	Rojas-Oropeza et al. [74]
FW	920	195.5	D	2 years	Marín-Muñiz et al. [7,8]
Mangroves		11.95	H	2 years	Chuang et al. [75]

* Only one sample collection.

Policy-based interest in carbon sequestration has increased recently, and wetland creation/restoration projects have high potential for carbon credits through soil carbon sequestered [76]. De la Peña et al. [77] evaluated in monetary terms the service of carbon store for “The Cienega Grande de Santa Marta” (the largest of the wetland areas located in Colombia), in which they found that the monetary valuation was between USD 87.76 and USD 591.41 for the area of mangroves, such valuation according to the carbon market established by the World Bank with the *Biocarbon found*. In addition, mangrove ecosystems have been described as sentinel-ecosystems in front of climate change impact [78].

With the works reviewed, it is clear that the number of publications and study sites on carbon stock and fluxes in Mexican forested wetlands is still very small. However, the values regarding carbon dynamic in forested wetlands are important to mitigate global warming. For this reason, it is necessary to increase research in this area, and enact laws that protect these important carbon sinks. The ability to sequester carbon of wetlands is being considered in national GHG emissions assessments and private initiatives as a potential source of revenue to manage carbon-balanced landscapes and pay for ecosystem services [65]. Data reported in this study for forested wetlands are necessary to use in global carbon budgets or carbon balance in the world.

### Key Points about the Role of Forested Wetlands for Climate Change Mitigation

- Considering the carbon sinks detected in the review, wetland soils as carbon pool are an innovative solution for climate change mitigation and adaptation at an international level.
- To guarantee the climate change mitigation by Forest-W, it is necessary to secure undrained wetland soils, rewet and restore drained wetlands and make a sustainable use.
- Promoting environmental education programs regarding ecosystem services of wetlands is a strategy to ensure the wetland conservation and its carbon sink function.
- It is necessary to extrapolate the role of wetlands in other climates that are likely to experience changes.
- Irrespective of uncertainties and the unique nature of implementing projects regarding carbon pool in wetlands to mitigate climate change, Forest-W are prime ecosystems for reforestation and restoration.

## 4. Conclusions

Carbon stock and carbon sequestration are an important ecosystem service that wetlands provide. With literature data, in this study, soils of mangroves, flooded palms and freshwater wetlands (Forest-W) from Mexican tropical regions have revealed through carbon stock and carbon sequestration values that such ecosystems play an important role in climate regulation. FW are the forested wetland soils with higher carbon stock reported, followed by mangrove, and flooded palms, indicating the relevance of conservation, creation, and reforestation of tropical Mexican wetlands to maintain and increase the carbon pool function. The data analysis on carbon sink in Mexican forested wetlands can help to inform practitioners in future decisions regarding sustainable projects and public policy (payment for environmental services), restoration, conservation, or creation projects. Moreover, the values reported for Mexican forested wetlands here can be used in carbon budgets/carbon balance around the world. Another important aspect to consider is the availability of public access to these studies or inventories carried out in Mexico and other regions and to create a public data set on the carbon inventory of the Mexican wetlands. Thus, more research in this matter is needed to estimate with more accuracy the current role of tropical wetlands in global carbon cycles

## Figures and Tables

**Table 1:** Carbon stock in Mexican forested wetland soils based on field studies.

Forested Wetland Type	Site (Municipality or Area, State)	Carbon Stock (kg C m^−2^)	Location in the Map (Figure 2)	Reference
Mangrove	Huimanguillo and Cárdenas, Tabasco	64.7	E	Moreno et al. [4]
Mangrove	Laguna de Términos, Campeche	25.2	G	Moreno-May et al. [21]
Mangrove	Carmen city, Campeche	11.7	G	Ceron-breton et al. [22]
Mangrove	Isla Pitaya, Quintana Roo.	15.7	I	Adame et al. [23]
Mangrove	La Encrucida, Biosphere Reserve, Chiapas	21.5	F	Adame et al. [24]
Mangrove	Pantanos de Centla, Tabasco and Campeche	45.8	E, G	Kauffman et al. [25]
Mangrove	Vega de Alatorre, Veracruz	22	D	Hernández et al. [26]
Mangroves	Alvarado, Veracruz	16	D	Moreno-Casasola et al. [27]
Mangrove	Tuxpan, Veracruz	14.7	D	Santiago [28]
Mangrove	Agua Brava Lagooon, Nayarit	4.2	C	Herrera-Silveira et al. [29]
Mangrove	Bahía Tóbari, Sonora	7.9	B	Bautista-Olivas et al. [30]
Mangrove	Cuyutlán, Colima	10.2	J	Herrera-Silveira et al. [29]
Mangrove	Nayarit	12	C	Valdés et al. [31]
Mangrove	La Paz Baja California	17.5	A	Ochoa-Gómez et al. [32]
Mangrove	Central coastal plain of Veracruz	37.5	D	Hernández and Junca-Gómez [33]
Mangrove	Paraíso Tabasco	20	E	Arias [34]
Mangrove	Península Yucatán	28.7	H	Gutiérrez-Mendoza and Herrera-Silveira [35]
Mangrove	Celestun, Yucatán	61.6	H	Herrera-Silveira et al. [36]
Mangrove	Nayarit	10	C	Valdés et al. [37]
Mangrove	Magdalena and Malandra bay. Baja California	22.5	A	Ezcurra et al. [38]
Mangrove	Sian Ka’an, Quintana Roo	45	I	Herrera-Silveira et al. [29]
Mangrove	Puerto Morelos, Yucatán	36	H	Herrera-Silveira et al. [29]
Mangrove	Aguiabampo, Sonora	3.5	B	Barreras-Apodaca et al. [39]
Mangrove	El Rabón, Nayarit	30	C	Castillo-Cruz and Rosa-Meza [40]
Mangrove	La Encrucijada, Chiapas	17.9	F	Barreras-Apodaca et al. [39]
Mangrove	Isla Arena, Campeche	30.5	G	Pech-Poot et al. [41]
Mangrove	Celestún, Yucatán	22.4	H	Pech-Poot et al. [41]
Mangrove	Cancún, Quintana Roo	26.4	I	Pech-Poot et al. [41]
Mangrove	La Encrucijada, Chiapas	6.3	F	Velázquez-Pérez et al. [42]
Freshwater	La Encrucida, Biosphere Reserve, Chiapas	9.5	F	Adame et al. [24]
Freshwater	Jamapa, Veracruz	39	D	Hernández et al. [26]
Freshwater	Alvarado, Veracruz	60	D	Moreno-Casasola et al. [27]
Freshwater	Tecolutla, Actopan, and Alto Lucero, Veracruz	45	D	Marín-Muñiz et al. [7]
Freshwater	Alto Lucero and Tecolutla, Veracruz	52	D	Campos et al. [43]
Freshwater	Tecolutla and Vega de Alatorre, Veracruz	35	D	Marín-Muñiz et al. [44]
Flooded Palm	Sian Ka’an, Quintana Roo	6.5	I	Alamilla, [45]
Flooded Palm	Alvarado, Veracruz	16	D	Moreno-Casasola et al. [27]
Flooded Palm	Jamapa, Veracruz	1.5	D	Sánchez [46]
